# Successful Inactivation of High-Consequence Pathogens in PrimeStore Molecular Transport Media

**DOI:** 10.3390/v17050639

**Published:** 2025-04-29

**Authors:** Briana Spruill-Harrell, Gregory Kocher, Maurice Boda, Kristen Akers, Denise Freeburger, Nicole Murphy, Jens H. Kuhn, Gerald Fischer, Irina Maljkovic Berry, Prabha Chandrasekaran, Jerry Torrison

**Affiliations:** 1 Integrated Research Facility at Fort Detrick, National Institute of Allergy and Infectious Diseases, National Institutes of Health, B-8200 Research Plaza, Fort Detrick, Frederick, MD 21702, USA; briana.spruill-harrell@nih.gov (B.S.-H.); gregory.kocher@nih.gov (G.K.); maurice.boda@nih.gov (M.B.); kristen.akers@nih.gov (K.A.); denise.freeburger@nih.gov (D.F.); nicole.murphy@nih.gov (N.M.); kuhnjens@mail.nih.gov (J.H.K.); prabhanih@gmail.com (P.C.); 2 Longhorn Vaccines and Diagnostics, LLC, Bethesda, MD 20814, USA; gwf@lhnvd.com

**Keywords:** high-consequence pathogen, inactivation, molecular transport medium, PrimeStore MTM, virus

## Abstract

Handling cultured isolates and clinical, environmental, or wildlife surveillance samples containing Risk Group 3 and 4 pathogens presents considerable biosafety challenges in minimizing human exposure during processing and transport. Safe handling typically requires high- or maximum-containment facilities, demanding substantial logistical planning and resources. We evaluated PrimeStore Molecular Transport Medium (PS-MTM), a guanidine-based solution created to kill pathogens and preserve nucleic acids at ambient temperatures, for inactivating Crimean-Congo hemorrhagic fever, eastern equine encephalitis, Ebola, Hendra, Japanese encephalitis, Lassa, Marburg, Nipah, Rift Valley fever, and West Nile viruses. To mimic diagnostic conditions, human whole blood spiked with any of these viruses was incubated with PS-MTM for 20-, 30-, or 60-min. Samples with titers up to 10^7^ PFU/mL exposed to PS-MTM at all time points resulted in complete loss of infectivity judged by plaque assays. A 30-min incubation provided a 50% safety margin over the minimum inactivation time and was used for quantification with the tissue culture infectious dose (TCID_50_) assay, enabling evaluation of PS-MTM’s activity for viruses that do or do not produce well-defined plaques. Results confirmed that PS-MTM inactivated all tested viruses at titers up to 10^7^ TCID_50_/mL, underscoring its reliability for enhancing biosafety in diagnostics, outbreak management, and surveillance.

## 1. Introduction

Risk Group 3 and 4 pathogens are associated with severe and often fatal diseases in humans and other animals. These pathogens pose significant biological threats, and many have the potential to cause pandemics. Even under controlled conditions, handling diagnostic samples containing these pathogens presents considerable biosafety risks, particularly during processing and transportation [1]. Safe handling of samples typically requires specialized high- (biosafety level 3 [BSL-3]) or maximum-containment (BSL-4) laboratories [2]. However, establishing and maintaining these facilities requires substantial logistical planning and financial resources, the latter of which can be challenging to sustain even in well-resourced regions [3]. Additionally, many diagnostic workflows rely on maintaining a cold chain from the point of sample collection to the laboratory to preserve sample integrity and to ensure accurate downstream analysis [4]. Implementing and sustaining such systems can be logistically demanding in well-resourced settings and present additional challenges in resource-limited regions. These complexities are particularly evident during active surveillance studies, which are critical for monitoring pathogen circulation in endemic and non-endemic areas [5].

PrimeStore Molecular Transport Medium (PS-MTM), the first Class II microbial nucleic acid storage and stabilization device cleared by the United States (U.S.) Food and Drug Administration (FDA) [6] offers a promising solution to these challenges. This medium is primarily composed of the chaotropic agent guanidine thiocyanate, which denatures proteins, lyses cells and virus particles and inactivates DNases and RNases to prevent nucleic acid degradation [7,8]. PS-MTM inactivates bacteria (e.g., *Bacillus subtilis*, *Escherichia coli*, *Mycobacterium tuberculosis*, *Pseudomonas aeruginosa*, and *Staphylococcus aureus*), fungi (e.g., *Candida albicans*, *Aspergillus brasiliensis*), and viruses (e.g., eastern equine encephalitis virus [EEEV]/Sindbis virus [SINV] chimera, human adenovirus 5, influenza A virus [FLUAV] H3N2 and H5N1, monkeypox virus [MPXV], and severe acute respiratory syndrome coronavirus 2 [SARS-CoV-2]) at ambient temperatures [8,9,10,11]. PS-MTM has been widely used for the collection, storage, and transport of samples in animal and clinical surveillance studies [12,13,14]. For example, the U.S. Walter Reed Army Institute of Research has used PS-MTM for genomic surveillance of SARS-CoV-2 in NATO/U.S. military camps in Afghanistan, including during the withdrawal of U.S. forces, a period marked by logistical disruptions and the collapse of the cold chain [13]. Similarly, the U.S. Department of Agriculture has used PS-MTM for surveillance of highly pathogenic avian FLUAV in wildlife [15]. Additionally, PS-MTM effectively stabilizes nucleic acids at room temperature, facilitating their use in molecular assays, including PCR and sequencing [8,13,15,16,17].

Although PS-MTM has been shown to be active against some viruses, the inactivation of most viruses listed on the National Institute of Allergy and Infectious Diseases (NIAID) Biodefense Pathogen List [18] remains unassessed [8,9,15]. Pandemic threats from high-consequence viruses (e.g., Crimean-Congo hemorrhagic fever virus [CCHFV], Ebola virus [EBOV], Hendra virus [HeV], Japanese encephalitis virus [JeV], Lassa virus [LASV], Marburg virus [MARV], Nipah virus [NiV], Rift Valley fever virus [RVFV], and West Nile virus [WNV]) remain a major concern. These pathogens are often endemic in resource-limited regions, where finite diagnostic, surveillance, cold chain, and containment infrastructure exacerbate their impact [5,19,20]. Coupled with high case-fatality rates, human-to-human transmission, and emerging zoonotic risks, these challenges present significant barriers to effective disease control. This underscores the importance of approaches that increase diagnostic accuracy, biosafety, and biosecurity for high-consequence pathogens, even in resource-limited settings.

In this study, we evaluated the effectiveness of PS-MTM in inactivating high-consequence pathogens from the virus families *Arenaviridae* (LASV), *Filoviridae* (EBOV, MARV, Reston virus [RESTV]), *Flaviviridae* (JeV, WNV), *Nairoviridae* (CCHFV), *Paramyxoviridae* (HeV, NiV), *Phenuiviridae* (RVFV), and *Togaviridae* (EEEV) using virus stocks available at the Integrated Research Facility at Fort Detrick (IRF-Frederick). We assessed the activity of PS-MTM in reducing viral infectivity using plaque assays and tissue culture infectious dose assays.

## 2. Materials and Methods

All work with infectious viruses was conducted in a maximum containment (BSL-4) laboratory.

### 2.1. Cells and Viruses

#### 2.1.1. Cells

Grivet (*Chlorocebus aethiops* (Linnaeus, 1758)) epithelial kidney Vero E6 cells (Catalog No. NR-596, BEI Resources, Manassas, VA, USA) and golden hamster (*Mesocricetus auratus* (Waterhouse, 1839)) kidney BHK-21 fibroblasts (Catalog No. CCL-10, American Culture Collection [ATCC], Manassas, VA, USA [ATCC]) were cultured at 37 °C with 5% carbon dioxide (CO_2_), whereas human adrenal cortex epithelial SW-13 cells (Catalog No. CCL-105, ATCC) were cultured at 37 °C without extraneous CO_2_. Vero E6 cells were maintained in Dulbecco’s Modified Eagle’s Medium (DMEM; Catalog No. 11995040, Gibco, Waltham, MA, USA), BHK-21 cells in Eagle’s Minimum Essential Media (EMEM; Catalog No. 30-2003, ATCC), and SW-13 cells in Leibovitz’s L-15 medium (Catalog No. 11415064, Gibco). All media were supplemented with 10% (*v*/*v*) heat-inactivated fetal bovine serum (FBS; Catalog No. F4135, Millipore Sigma, Burlington, MA, USA) without antibiotics (in accordance with institutional standard operating procedures to maintain aseptic conditions and enable contamination monitoring). Cells were seeded in T25 flasks or T75 flasks for passaging in tissue culture, 6-well plates for plaque assays, and 96-well plates for tissue culture infectious dose (TCID_50_) assays. Cells were incubated for 24 h prior to use.

#### 2.1.2. Viruses

Crimean-Congo hemorrhagic fever virus isolate IbAr10200 (CCHFV; Internal Reference No. IRF0414) was obtained from the World Reference Collection of Emerging Viruses and Arboviruses (WRCEVA) at the University of Texas Medical Branch at Galveston (UTMB), Galveston, TX, USA. Eastern equine encephalitis virus isolate FL93-939 (EEEV; Internal Reference No. IRF0539), isolated in 1993 from a pool of black-tailed mosquitoes (*Culiseta melanura* (Coquillett, 1902)) in Florida, USA, was obtained from BEI Resources (Item No. NR-41567). Ebola virus variant Makona isolate C05 (EBOV; Internal Reference No. IRF0137), isolated in 2014 from the blood of a patient [21], was provided by the Public Health Agency of Canada (PHAC), Ottawa, ON, Canada [21]. Reston virus strain Philippines89-AZ-1435 (RESTV; Internal Reference No. IRF0195) was provided by the U.S. Army Medical Research Institute of Infectious Diseases (USAMRIID; Fort Detrick, Frederick, MD, USA). Hendra virus (HeV; Internal Reference No. IRF0359) was provided by USAMRIID. Japanese encephalitis virus isolate Nakayama (JEV), originally isolated in 1935 from the cerebrospinal fluid of a 6-year-old patient, was obtained from BEI Resources (Item No. NR-92). Lassa virus isolate SN61 (LASV; Internal Reference No. IRF0562) was provided by the National Microbiology Laboratory (NML), Winnipeg, Manitoba, Canada. Marburg virus variant Mt. Elgon isolate Musoke (MARV; Internal Reference No. IRF0296) was provided by USAMRIID. Nipah virus Bangladesh isolate 810398 (NiV-B, Internal Reference No. IRF0284), isolated in 2004 from an oropharyngeal swab from a patient in Bangladesh [22], was provided by the Special Pathogens Branch of the U.S. Centers for Disease Control and Prevention (CDC), Atlanta, GA, USA. NiV Malaysia (NiV-M; Internal Reference No. IRF0523) was provided by USAMRIID. Rift Valley fever virus isolate ZH501 (RVFV) was obtained from BEI Resources (Item No. NR-37378). Venezuelan equine encephalitis TC83 virus acquired from BEI Resources (NR-63). West Nile virus isolate 385-99 (WNV) was obtained from WRCEVA.

Table A1 lists all viruses and cell lines used for infection or virus propagation.

### 2.2. Virus Inactivation with PS-MTM

**Scheme 1:**
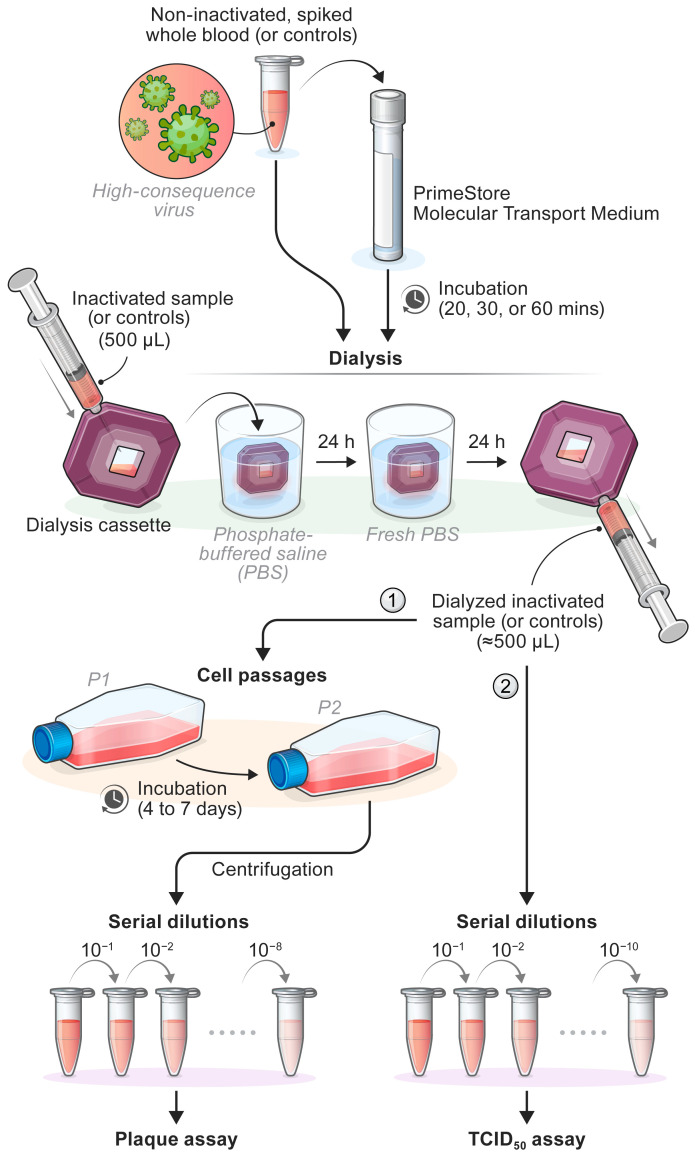
Inactivation Procedure for High-Consequence Pathogens using PrimeStore Molecular Transport Media (PS-MTM). ① For safety testing, dialyzed samples were subjected to two blind serial passages in naïve Vero E6 cells prior to serial dilution and plaque assay. ② For TCID_50_ assays, serial passaging was not performed. Samples were immediately serial-diluted and assessed.

To better simulate diagnostic conditions, the inactivation process was evaluated on human whole blood spiked with test virus at titers higher or comparable to those found in clinical samples [23,24,25,26,27]. Whole blood from healthy human donors (BioIVT, Westbury, NY, USA) was spiked with CCHFV, EBOV, RESTV, EEEV, HeV, JEV, LASV, MARV, NiV-B, NiV-M, RVFV, or WNV. Non-spiked whole blood and non-spiked whole blood treated with PS-MTM served as negative controls. Samples were treated with PS-MTM (Catalog No. LH-PS-MTM-12, Longhorn Vaccines and Diagnostics, Bethesda, MD, USA) following the manufacturer’s instructions. One part of spiked whole blood or negative control was mixed with three parts of PS-MTM and incubated at room temperature for 20-, 30-, or 60-min (Scheme 1). For each condition, samples derived from the same source material were inactivated in triplicate for the plaque assay and in duplicate for the TCID_50_ assay.

### 2.3. Evaluation of PS-MTM Removal by Dialysis

To assess virus loss during the removal of the inactivation reagent, virus stocks from representative virus families were dialyzed prior to inactivation with PS-MTM. Testing was performed using VEEV (*Togaviridae*), RVFV (*Phenuiviridae*), EBOV (*Filoviridae*), LASV (*Arenaviridae*), HeV (*Paramyxoviridae*), and WNV (*Flaviviridae*). For each virus, three 500-µL aliquots were dialyzed in phosphate-buffered saline (PBS) using Slide-A-Lyzer dialysis cassettes with a molecular weight cutoff of 20 kDa (Catalog No. 66005, Thermo Fisher Scientific, Waltham, MA, USA). Dialysis was performed at room temperature for 24 h, after which PBS was replaced with fresh buffer, and dialysis continued for an additional 24 h. The final volume (≈500 µL) from each replicate was collected and used to inoculate cells for plaque assays to evaluate viral titers after dialysis. Undialyzed aliquots from each virus were used to inoculate cells for plaque assays for viral titer comparison. Comparisons of paired data (pre-dialysis vs. post-dialysis) were performed using the Wilcoxon matched-pairs signed-rank test (two-tailed) in GraphPad Prism (version 10.2.0 for Windows, GraphPad Software, Boston, MA, USA).

### 2.4. Sample Dialysis and Serial Passaging for Safety Testing

After evaluating potential virus loss during dialysis, inactivated samples, the positive control (non-inactivated spiked whole blood), and PS-MTM alone were dialyzed in PBS to remove residual detergent and minimize cytotoxicity. As described above, each 500 µL sample was dialyzed using Slide-A-Lyzer dialysis cassettes with a molecular weight cutoff of 20 kDa. Dialysis was performed at room temperature for 24 h, followed by PBS replacement and an additional 24 h dialysis. The final volume (≈500 µL) was collected and used to inoculate cells for the validation of chemical inactivation via plaque assay or TCID_50_ assay (Scheme 1).

For safety testing by plaque assay, dialyzed samples underwent two blind serial passages on naïve cultured Vero E6 cells. In contrast, serial passaging was not required for the TCID_50_ assay. Cells were incubated for 4 (EEEV-FL93-939, HeV, RVFV-ZH501, and WNV-385-99), 6 (LASV), or 7 d (EBOV). Following the first passage, the cell culture supernatants and monolayers from each T25 flask were collected and clarified by centrifugation. The entire clarified supernatants were then transferred to fresh T75 flasks containing Vero E6 cells for the second passage. These were incubated under the same conditions, and the resulting clarified supernatants (900 µL) were used for plaque assays (Scheme 1).

### 2.5. Plaque Assays

Vero E6 cells were seeded in 6-well plates 24 h before use and subsequently transferred to the BSL-4 laboratory. A 2.5% Avicel 591 overlay was prepared by mixing Avicel PH-101 cellulose resin (Catalog No. 11365-1KG, Millipore Sigma) with 2× Temin’s Modified Eagle Medium (Catalog No. 11935046, Gibco, Waltham, MA, USA), supplemented with 4% heat-inactivated FBS (*v*/*v*) and 2% penicillin-streptomycin (*w*/*v*) in a 1:1 ratio to achieve a final 1X concentration. Samples were serially diluted 10-fold in DMEM supplemented with 2% heat-inactivated FBS (*v*/*v*) and 1.06% penicillin-streptomycin (*w*/*v*) with a dilution range of 10^−1^–10^−8^ (Scheme 1). Cell culture supernatants from the 6-well plates were removed, and 300 μL of diluted samples were added to wells in triplicate. Plates were incubated at 37 °C with 5% CO_2_ for 1 h and gently rocked every 15 min. After incubation, 2 mL of Avicel 591 overlay were added to each well, and plates were incubated for 4 (EEEV, HeV, RVFV, and WNV), 6 (LASV), or 7 d (EBOV). After incubation, overlays were discarded, and cells were stained for 30 min with a 0.2% crystal violet solution (2% Gentian Violet [Catalog No. 3233, Ricca Chemical Company, Arlington, TX, USA] prepared in 10% neutral buffered formalin [Catalog No. 16004-126, VWR, Radnor, PA, USA]). Plaques were visually counted immediately after staining, and their numbers were recorded.

### 2.6. TCID_50_ Assays

Vero E6, BHK-21, and SW-13 cells were seeded in 96-well plates 24 h before use and subsequently transferred to the BSL-4 laboratory. Ten-fold serial dilutions of the inactivated samples, as well as positive and negative controls, were prepared using a cell culture medium (as described in the Section 2.1), supplemented with 2% heat-inactivated FBS (*v*/*v*). Dilutions (range of 10^−1^–10^−10^) were added to wells in triplicate (100 µL per well) for each technical replicate (Scheme 1). Non-spiked whole blood and non-spiked whole blood treated with PS-MTM were included on each plate.

Plates were incubated at 37 °C with 5% CO_2_ for 1 h and gently rocked every 15 min; SW-13 cells were incubated at 37 °C without extraneous CO_2_. After incubation, 100 μL of DMEM, supplemented with 2% heat-inactivated FBS, were added to wells. Then, plates were incubated for 4 (JEV, NiV-B, and NiV-M), 5 (HeV), 6 (RVFV), 7 (CCHFV), 10 (EBOV and MARV), or 13 d (RESTV).

Following incubation, cells were stained with 100 μL of 0.4% crystal violet solution for 30 min at room temperature. Plates were then washed twice with deionized water and air dried. TCID_50_s were calculated using the Reed–Munch method [28].

### 2.7. Statistical Analysis

Statistical comparisons of control and treatment groups were not performed due to the absence of detectable variability in plaque assay or TCID_50_ values for treatment groups, which consistently did not result in cytopathic effects (CPE) or infectivity.

## 3. Results

### 3.1. Plaque Assay Confirms That PS-MTM Effectively Inactivates High-Consequence Pathogens After Incubation for 20, 30, and 60 Min

To determine the time required for PS-MTM to fully inactivate high-consequence pathogens and evaluate its effectiveness in reducing viral infectivity, representative viruses from the *Arenaviridae*, *Filoviridae*, *Flaviviridae*, *Paramyxoviridae*, *Phenuiviridae*, and *Togaviridae* families were treated with PS-MTM for 20-, 30- and 60-min, and viral infectivity was subsequently assessed using plaque assays. Inactivation was evaluated in the presence of whole blood to better simulate diagnostic sample conditions. Prior to each inactivation, the initial viral concentration was determined to establish the maximum starting titer (Table 1). Virus-spiked whole blood samples were then treated with PS-MTM, whereas non-spiked whole blood negative controls and spiked whole blood positive controls were left untreated. All samples and controls were dialyzed before being assessed for infectivity by plaque assay.

All tested samples were fully inactivated following PS-MTM treatment. No viable virus was detected at 20-, 30-, or 60-min post-inactivation for any of the viruses tested (Table 2). For the positive controls, CPE was evident; however, for LASV-, no CPE was observed, despite detectable infectivity by plaque assay.

### 3.2. Dialysis Does Not Significantly Reduce Virus Titer

To determine whether dialysis affected viral recovery, high-titer virus stocks from each representative family were dialyzed in PBS prior to inactivation. Plaque assays performed on pre- and post-dialysis samples showed minimal reduction in virus titers, indicating that the dialysis procedure itself did not contribute to a significant loss of viral infectivity (Table A2). Moreover, no cytopathic effects were observed in wells inoculated with dialyzed PS-MTM controls, indicating minimal residual toxicity (Table 2). These findings support the validity of subsequent inactivation assays conducted after dialysis.

### 3.3. TCID_50_ Assay Confirms Complete Inactivation of High-Consequence Pathogens by PS-MTM

For viruses, such as CCHFV that do not produce observable plaques [29] or HeV and NiV that may produce poorly defined plaques due to syncytia formation [30], TCID_50_ assays were used as an alternative method to quantify infectious virus particles. Furthermore, TCID_50_ assays were used with other viruses at the IRF-Frederick known to form well-defined plaques to comprehensively evaluate its utility in titrating high-consequence pathogens (EBOV, RESTV, MARV, JEV, RVFV). Although a plaque assay was successfully performed on LASV, the TCID_50_ assay was not able to be used for this virus due to the absence and/or inconsistency of CPE, which is required as an endpoint for the TCID_50_ assay. Thus, we relied on the plaque assay to quantify the viral infectivity of LASV.

The ability of PS-MTM to fully reduce viral infectivity was evaluated after a 30-min incubation. This duration was chosen based on previous studies demonstrating that a 30-min incubation effectively inactivated a representative subset of viruses and provided a 50% safety margin over the minimum inactivation time determined by plaque assay (Table 2). Although not all viruses were tested at 20 and 60 min, the 30-min incubation was selected to standardize the protocol and ensure consistency across experiments.

For these subsequent experiments, inactivation was assessed directly by TCID_50_ assay following dialysis rather than by serial passaging and plaque assay, which had already been used to confirm complete inactivation for representative viruses from each viral family. An exception was CCHFV, the representative member of the *Nairoviridae* family, for which serial passaging was not performed.

**Figure 1:**
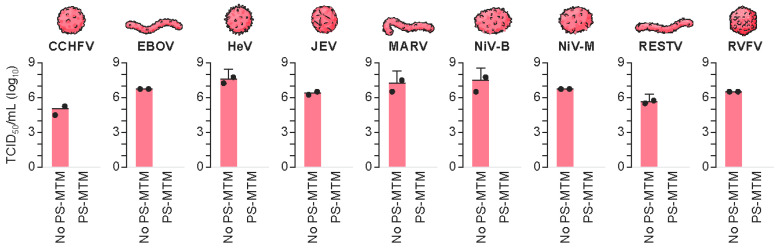
Inactivation of High-Consequence Pathogens by PrimeStore Molecular Transport Media (PS-MTM) at 30 min. Bar graphs represent the mean log_10_ TCID_50_/mL from duplicates, with the error bars indicating the 95% confidence intervals. CCHFV, Crimean-Congo hemorrhagic fever virus; EBOV, Ebola virus; HeV, Hendra virus; JEV, Japanese encephalitis virus; MARV, Marburg virus; NiV-B, Nipah virus Bangladesh; NiV-M, Nipah virus Malaysia; RESTV, Reston virus; RVFV, Rift Valley fever virus; TCID_50_, tissue culture infectious dose.

Results from the TCID_50_ assay showed that PS-MTM fully inactivated CCHFV, EBOV, RESTV, HeV, JEV, MARV, NiV-B, NiV-M, and RVFV in whole blood within 30 min (Figure 1). Post-inactivation, no CPE was observed, and virus titers were reduced by 5–7 log units. Although the observed inactivation of CCHFV supports PS-MTM’s broader efficacy, further validation using serial passaging is needed to fully confirm inactivation for nairovirids.

## 4. Discussion

Handling high-consequence pathogens in a BSL-2 environment requires an inactivation step to ensure biosafety [31]. Several methods have been described for enveloped viruses, including heat inactivation, ultraviolet radiation, lipid solvents, and guanidine-based lysis solutions [32,33,34,35,36,37]. The choice of method depends on whether the goal is to preserve protein structure (e.g., for serological assays) or to maintain nucleic acid integrity (e.g., for molecular assays). PS-MTM is a guanidine-thiocyanate-based solution with ethanol and *N*-lauroylsarcosine sodium salt, specifically designed to inactivate pathogens while stabilizing nucleic acids [7]. Traditional guanidine-thiocyanate-based solutions, such as AVL buffer (Qiagen) and TRIzol LS reagent (Invitrogen), are widely used for pathogen inactivation and RNA extraction but differ in long-term stabilization [32]. AVL buffer, particularly when combined with ethanol, effectively inactivates pathogens but is not suitable for long-term storage, requiring refrigeration or freezing (−20 or −80 °C) to prevent RNA degradation [38,39]. Similarly, TRIzol LS is not designed for long-term stability at ambient temperature and requires frozen storage [40]. In contrast, PS-MTM stabilizes RNA and DNA at ambient temperatures for up to 30 d, thereby eliminating the need for refrigeration or freezing [15,41,42].

This study demonstrates that PS-MTM effectively inactivates high-consequence representative pathogens from the virus families *Arenaviridae*, *Filoviridae*, *Flaviviridae*, *Nairoviridae*, *Paramyxoviridae*, *Phenuiviridae*, and *Togaviridae*, within 30 min of incubation in whole blood. Inactivation was confirmed at the highest tested titers (10^4^–10^7^ TCID_50_/mL or PFU/mL) by TCID_50_ and plaque assays. These titers were comparable to or exceed those reported in clinical samples for some of the pathogens, such as EBOV (up to 1 × 10^7^ PFU/mL in serum) [23,24], LASV (up to 2.5 × 10^5^ PFU/mL in cerebrospinal fluid after serial passage) [27], MARV (up to 1 × 10^7^ TCID_50_/mL in whole blood and bodily fluids, based on cycle threshold (Ct) conversions) [25], and RVFV (up to 1 × 10^6^ TCID_50_/mL in whole blood) [26]. These findings highlight PS-MTM’s ability to inactivate viruses even at concentrations reflective of severe infections. For some pathogens, such as EEEV, HeV, JEV, and NiV, data on viral loads in human clinical samples are scarce, limiting direct comparisons. Most available information comes from in vitro and/or in vivo experimental infection studies [43,44,45,46,47], whereas, for JEV, diagnostics primarily relies on detecting virus-specific antibodies or RNA rather than quantifying viral titers [48,49]. For CCHFV, viral loads are typically reported in copies per mL rather than infectious titers, with values reaching up to 1.3 × 10^10^ copies per mL in sera, depending on disease severity [50,51]. Since only a fraction of virus particles are replication-competent, these values likely overestimate infectious titers, making direct comparisons challenging [52]. Nonetheless, the infectious titers measured in this study likely represent biologically relevant levels, reinforcing the utility of PS-MTM in diagnosis and surveillance. Future studies could incorporate low-titer spiked controls to empirically determine assay sensitivity.

In addition to demonstrating effective viral inactivation, an important consideration for chemical agents such as PS-MTM is their potential cytotoxicity, which can interfere with the accurate assessment of infectivity. PS-MTM contains chaotropic agents that are cytotoxic to cells if not diluted or adequately removed [8,15]. To reduce cytotoxicity prior to infectivity assays, we used a two-step dialysis procedure in which virus-containing samples were placed in dialysis cassettes and submerged in PBS. Consequently, low molecular weight compounds, such as residual inactivation reagents, could diffuse out through the membrane while virus particles were retained. The absence of CPE in the dialyzed whole-blood and PS-MTM controls, combined with the ability to detect viruses in non-inactivated and pre-dialyzed samples, supports the effectiveness of this purification method. Previous studies have demonstrated that cytotoxic components can be effectively removed using methods such as dialysis, dilution of the inactivating reagent, extensive washing steps, or size-exclusion spin column filtration [10,15,36,53,54,55,56]. Herein, the dialysis method used aligns with these studies and with safety testing procedures at our facility. To expand PS-MTM use for diagnostic or research applications, future studies could directly compare purification methods and quantify residual cytotoxicity. Additionally, although our current experimental design confirmed the removal of cytotoxicity and the ability of cells to support virus replication post dialysis, we did not include a control in which infectious virus was spiked into PS-MTM-treated whole blood samples after dialysis. This control would help confirm that residual PS-MTM components, even after purification, did not interfere with virus entry or replication. Including this type of control in future experiments would further strengthen confidence in assay sensitivity.

Overall, this study demonstrates PS-MTM’s ability to inactivate high-consequence pathogens, but several areas warrant further investigation to fully understand its potential and optimize its use. For example, future studies should assess PS-MTM’s inactivation efficacy against additional Risk Group 3 and 4 priority pathogens, including bacteria, fungi, and toxins. Additionally, the inclusion of more diverse sample matrices, such as cerebrospinal fluid, oral swabs, seminal fluid, or tissue homogenates, would provide greater insight into its versatility.

One key area for future research is the evaluation of PS-MTM’s potency. Determining the minimum inactivation concentration (MIC) of PS-MTM or the concentration required to inactivate 50% of viral infectivity (IC_50_) in samples with higher viral titers would provide valuable insights into the efficiency of PS-MTM under varying conditions. This is particularly critical for optimizing the sample-to-PS-MTM ratio in scenarios in which precious or cultured samples contain limited copies of target genetic material that may fall below detectable levels if diluted. At the current 1:3 ratio, the target material may be considerably diluted. Furthermore, the identification of the maximum viral concentration that can be completely inactivated at the recommended sample-to-PS-MTM ratio and exposure time would strengthen its applicability in both clinical and research settings.

Another important focus should be the assessment of how long preserved nucleic acids maintain integrity at ambient temperatures for sequencing or other advanced applications. Additionally, the stability of host genetic material in PS-MTM-treated samples presents an important avenue for exploration. Investigating whether host nucleic acids remain intact could enable the use of inactivated samples for RNA sequencing and facilitate the identification of host-specific genomic markers associated with specific pathologies. This would expand the potential applications of PS-MTM beyond pathogen detection to include studies on host-pathogen interactions and disease mechanisms. These studies would not only establish PS-MTM’s broad applicability but also optimize its usage across diverse diagnostic and research workflows.

## 5. Conclusions

The effective inactivation of representative high-consequence pathogens from the *Arenaviridae*, *Filoviridae*, *Flaviviridae*, *Nairoviridae*, *Paramyxoviridae*, *Phenuiviridae*, and *Togaviridae* families by PS-MTM has significant implications for public health, biosafety, diagnostics, and research. By mitigating the risk of accidental exposure during the handling and transportation of high-risk biological samples, PS-MTM enhances laboratory safety and facilitates safer diagnostic workflows. This capability is particularly beneficial in field studies and resource-limited settings, where high- or maximum-containment facilities may not be accessible. Furthermore, PS-MTM contributes to advancing global surveillance of high-consequence pathogens, strengthening pandemic preparedness and supporting infectious disease research. Enabling safer diagnostic practices also accelerates the development of countermeasures against potential bioterrorism threats and improves outbreak response capabilities.

## Figures and Tables

**Table 1:** Maximum starting viral titer as determined by plaque assay.

Virus Family	Virus ^1^	Viral Titer (PFU/mL) ^2^			
		R1	R2	R3	Average
*Arenaviridae*	LASV	9.33 × 10^6^	9.00 × 10^6^	1.23 × 10^7^	1.06 × 10^7^
*Filoviridae*	EBOV	1.87 × 10^7^	1.80 × 10^7^	2.23 × 10^7^	1.97 × 10^7^
*Flaviviridae*	WNV	7.67 × 10^6^	1.10 × 10^7^	1.03 × 10^7^	9.67 × 10^6^
*Paramyxoviridae*	HeV	1.17 × 10^7^	1.00 × 10^7^	1.07 × 10^7^	1.08 × 10^7^
*Phenuiviridae*	RVFV	1.67 × 10^6^	1.33 × 10^6^	1.70 × 10^6^	1.57 × 10^6^
*Togaviridae*	EEEV	6.67 × 10^7^	7.67 × 10^7^	9.00 × 10^7^	7.78 × 10^7^

^1^ Viruses were spiked into healthy human whole blood. ^2^ All samples were plated in triplicate for plaque assays. “R” indicates a replicate, with the accompanying number specifying the replicate identifier. EBOV, Ebola virus; EEEV, eastern equine encephalitis virus; HeV, Hendra virus; LASV, Lassa virus; RVFV, Rift Valley fever virus; WNV, West Nile virus.

**Table 2:** Viral titers of PS-MTM-inactivated samples and controls obtained by plaque assay.

Virus Family	Virus ^1^	Average Titer (PFU/mL) ^2^			
		Inactivation Time			
		NA	20 min	30 min	60 min
NA	NA (Whole blood control)	0	NA	NA	NA
NA	NA (PS-MTM control)	0	NA	NA	NA
*Arenaviridae*	LASV	7.56 × 10^5^	0	0	0
*Filoviridae*	EBOV	6.89 × 10^6^	0	0	0
*Flaviviridae*	WNV	9.22 × 10^6^	0	0	0
*Paramyxoviridae*	HeV	5.56 × 10^6^	0	0	0
*Phenuiviridae*	RVFV	8.67 × 10^4^	0	0	0
*Togaviridae*	EEEV	1.72 × 10^7^	0	0	0

^1^ Viruses were spiked into healthy human whole blood. Controls included untreated samples. Negative controls consisted of dialyzed whole blood and PS-MTM-only controls. Positive controls consisted of whole blood spiked with virus. ^2^ All samples were plated in triplicate for plaque assays. EBOV, Ebola virus; EEEV, eastern equine encephalitis virus; HeV, Hendra virus; LASV, Lassa virus; NA, not applicable; PS-MTM, PrimeStore Molecular Transport Media; RVFV, Rift Valley fever virus; WNV, West Nile virus.

## Data Availability

All raw data are available upon request.

## Abbreviations

The following abbreviations are used in this manuscript:

ATCC	American Type Culture Collection
BSL	biosafety level
CCHFV	Crimean-Congo hemorrhagic fever virus
CDC	Centers for Disease Control and Prevention
CO_2_	carbon dioxide
CPE	cytopathic effect
Ct	cycle threshold
DMEM	Dulbecco’s Modified Eagle’s Medium
EBOV	Ebola virus
EEEV	Eastern equine encephalitis virus
EMEM	Eagle’s Minimum Essential Media
FBS	fetal bovine serum
FDA	Food and Drug Administration
FLUAV	influenza A virus
HeV	Hendra virus
IC_50_	concentration required to inactivate 50% of viral infectivity
IRF-Frederick	Integrated Research Facility at Fort Detrick
JEV	Japanese encephalitis virus
LASV	Lassa virus
MARV	Marburg virus
MIC	minimum inactivation concentration
MPXV	monkeypox virus
MRSA	methicillin-resistant *Staphylococcus aureus*
NIAID	National Institute of Allergy and Infectious Diseases
NiV	Nipah virus
NiV-B	Nipah virus Bangladesh
NiV-M	Nipah virus Malaysia
NML	National Microbiology Laboratory
PBS	phosphate-buffered saline
PHAC	Public Health Agency of Canada
qPCR	real-time polymerase chain reaction
RESTV	Reston virus
RVFV	Rift Valley fever virus
SARS-CoV-2	severe acute respiratory syndrome coronavirus 2
SINV	Sindbis virus
TCID_50_	tissue culture infectious dose
U.S.	United States
USAMRIID	U.S. Army Medical Research Institute of Infectious Diseases
USDA	U.S. Department of Agriculture
UTMB	University of Texas Medical Branch at Galveston
WNV	West Nile virus
WRCEVA	World Reference Collection of Emerging Viruses and Arboviruses

## Appendix A

**Table A1:** List of viruses used in the study and the cell lines used for infection or propagation.

Virus	Strain/Variant/Isolate	Cell Line	Reference GenBank Accession Number
CCHFV	IbAr10200	SW-13	PQ463984, PQ463983, PQ463982
EBOV	Makona-C05	Vero E6	KX000400
RESTV	Philippines89-AZ-1435	Vero E6	KY008770
EEEV	FL93-939	Vero E6	EF151502
HeV	unnamed	Vero E6	AF017149
JEV	Nakayama	BHK-21	EF571853
LASV	SN61	Vero E6	MZ169798, MZ169799
MARV	Musoke	Vero E6	AY430365
NiV-M	IRF523	Vero E6	PQ463988
NiV-B	810398	Vero E6	MK673564
RVFV	ZH501	Vero E6	PQ463985, PQ463986, PQ463986
WNV	385-99	Vero E6	AY842931

CCHFV, Crimean-Congo hemorrhagic fever virus; EBOV, Ebola virus; EEEV, eastern equine encephalitis virus; HeV, Hendra virus; JEV, Japanese encephalitis virus; LASV, Lassa virus; MARV, Marburg virus; NiV-B, Nipah virus Bangladesh; NiV-M, Nipah virus Malaysia; RESTV, Reston virus; RVFV, Rift Valley fever virus; WNV, West Nile virus.

**Table A2:** Viral titer loss during inactivation reagent removal.

Samples	Average Titer (Log_10_ PFU/mL)	Reduction from Initial Titer (Log_10_ PFU/mL)	Sum of Signed Ranks (*p*-Value) ^1^
EBOV initial titration	7.32	N/A	N/A
EBOV after dialysis 1	7.25	0.07	−6.00 (*p* = 0.25)
EBOV after dialysis 2	7.23	0.09	−6.00 (*p* = 0.25)
EBOV after dialysis 3	7.24	0.08	−6.00 (*p* = 0.25)
HeV initial titration	6.95	N/A	N/A
HeV after dialysis 1	7.01	−0.06 *	6.00 (*p* = 0.25)
HeV after dialysis 2	6.98	−0.03 *	3.00 (*p* = 0.50)
HeV after dialysis 3	6.90	0.05	−4.00 (*p* = 0.50)
LASV initial titration	6.92	N/A	N/A
LASV after dialysis 1	6.89	0.03	−1.00 (*p* > 0.99)
LASV after dialysis 2	6.82	0.10	−6.00 (*p* = 0.25)
LASV after dialysis 3	6.72	0.20	−6.00 (*p* = 0.25)
RVFV initial titration	6.81	N/A	N/A
RVFV after dialysis 1	6.58	0.23	−6.00 (*p* = 0.25)
RVFV after dialysis 2	6.61	0.20	−6.00 (*p* = 0.25)
RVFV after dialysis 3	6.59	0.22	−6.00 (*p* = 0.25)
VEEV initial titration	8.87	N/A	N/A
VEEV after dialysis 1	8.85	0.02	−4.00 (*p* = 0.50)
VEEV after dialysis 2	8.78	0.09	−6.00 (*p* = 0.25)
VEEV after dialysis 3	8.83	0.03	−2.00 (*p* = 0.75)
WNV initial titration	7.96	N/A	N/A
WNV after dialysis 1	7.98	−0.02 *	1.00 (*p* > 0.99)
WNV after dialysis 2	7.83	0.13	−6.00 (*p* = 0.25)
WNV after dialysis 3	7.82	0.14	−6.00 (*p* = 0.25)

* Negative reduction indicates an increase in titer. ^1^ Wilcoxon matched-pairs signed rank test statistic and two-tailed *p*-value are reported. Each group was compared to the initial viral titers prior to dialysis. EBOV, Ebola virus; HeV, Hendra virus; LASV, Lassa virus; RVFV, Rift Valley fever virus; VEEV, Venezuelan equine encephalitis virus; WNV, West Nile virus.
